# Origin of the *Eleusine indica* population on the active volcanic Nishinoshima Island, Japan

**DOI:** 10.1007/s10265-026-01738-9

**Published:** 2026-08-07

**Authors:** Koutaro Maeda, Takaki Aihara, Kentaro Uchiyama, Mengying Cai, Widiyatno, Takashi Kamijo, Yoshihiko Tsumura

**Affiliations:** 1 Graduate School of Life and Environmental Sciences, University of Tsukuba, Tsukuba, Ibaraki 305-8572 Japan; 2 Institute of Life and Environmental Sciences, University of Tsukuba, Tsukuba, Ibaraki 305-8572 Japan; 3 Department of Forest Molecular Genetics and Biotechnology, Forestry and Forest Products Research Institute, Tsukuba, Ibaraki 305-8687 Japan; 4 Faculty of Forestry, Gadjah Mada University, Bulaksumur, Yogyakarta, 55281 Indonesia

**Keywords:** Island, Long-distance gene flow, Origin, Seabirds, Seed dispersal

## Abstract

Nishinoshima Island, located approximately 1,000 km south of mainland Japan and 130 km from the Ogasawara Islands, emerged as a new island due to volcanic activity in 1973. On this isolated island, grasses such as *Eleusine indica* and *Portulaca oleracea* have been observed growing, though their routes of invasion remain unknown. Numerous seabirds have been spotted inhabiting and breeding around Nishinoshima Island, potentially contributing to the spread of these plants without any possibility of anthropogenic influence. The aim of this study was to trace the origin of the *E. indica* population on Nishinoshima Island using genetic methods. We collected samples of *E. indica* from Nishinoshima Island, mainland Japan, the Ryukyu Islands, the Ogasawara Islands, and other locations. In this study, we utilized two types of DNA with different inheritance modes: chloroplast DNA (cpDNA) and nuclear DNA. For cpDNA, five polymorphic regions were analyzed, while genetic diversity and structure were also assessed for nuclear DNA using MIG-seq (Multiplexed inter-simple sequence repeat (ISSR) genotyping by sequencing), a method for detecting nucleotide polymorphisms with next-generation sequencing technology. Genetic diversity analysis revealed high genetic diversity in the mainland Japan population. Additionally, cpDNA analysis confirmed the presence of the Nishinoshima haplotype on several islands, including Ogasawara Island. Nuclear DNA analysis showed a close relationship between the Nishinoshima population and those on Ogasawara, Hachijojima, Okinawa and Indonesia. Given instances of seabirds carrying tiny seeds over long distances, it is plausible that *E. indica* seeds were transported from distant locations by migrating birds. Based on the genetic diversity of *E. indica* on Nishinoshima, it was considered that this population originated from a single or a few seed as a result of an extremely rare seed dispersal event.

**Supplementary Information:**

The online version contains supplementary material available at https://doi.org/10.1007/s10265-026-01738-9.

## Introduction

When organisms colonize previously abiotic environments, ecological communities gradually assemble through a series of dispersal, establishment, and persistence processes. Elucidating the mechanisms underlying these early stages of colonization provides critical insights into ecosystem formation and island biogeography (Magnússon et al. 2022; Stuessy et al. 2022). However, direct observation of primary colonization events is rare, as it requires exceptional geological circumstances and timely biological surveys. In Japan, volcanic eruptions occasionally generate new islands, offering unique opportunities to investigate initial biological colonization and subsequent ecosystem development under well-defined temporal and spatial conditions. Nishinoshima, located approximately 130 km northwest of the Ogasawara Islands, represents one such case. Initially discovered in 1702 (referred to as “Old Nishinoshima”), the island remained volcanically inactive until 1973, when renewed activity led to the emergence of a new landmass and its subsequent merger with the original island (Mori et al. 2020). Further eruptions beginning in 2013 completely buried the pre-existing land surface beneath fresh volcanic deposits, effectively resetting the terrestrial environment and creating a biologically pristine island system.

In September 2019, during a temporary cessation of eruptive activity, the Nishinoshima Comprehensive Scientific Research Survey Team (organized by the Ministry of the Environment) conducted a multidisciplinary investigation of the island (Kamijo et al. 2020). These surveys encompassed geological and volcanic features as well as biotic components, including birds, insects, intertidal organisms, and plants (Homma et al. 2025; Noda et al. 2025). Three plant species, *Portulaca oleracea*, *Echinochloa crus-galli*, and *Eleusine indica*, were identified barely surviving within a residual 0.5-ha portion of Old Nishinoshima, and specimens were collected for analysis. Although all three species were subsequently eradicated by renewed volcanic activity by 2021, the preserved samples constitute invaluable material for examining early plant colonization processes on newly formed islands. Of particular significance, *E. indica* specimens were obtained in sufficient quantity to enable detailed ecological and genetic investigations.

The objective of this study is to elucidate the initial stages of plant immigration and ecosystem establishment on a newly formed, isolated volcanic island using *E. indica* as a model organism. *Eleusine indica* is a cosmopolitan grass with small, easily dispersed seeds often facilitated by avian vectors (Ridley 1930; Shmida & Ellner 1983), making it a suitable system for examining long-distance dispersal and founder events. By integrating maternally inherited chloroplast DNA markers with genome-wide nuclear SNPs obtained through MIG-seq, we assess both historical seed-mediated dispersal and contemporary population structure. Using comparative sampling from Nishinoshima, the nearby Ogasawara Islands, and adjacent continental regions, we aim to identify the origin of the Nishinoshima *E. indica* population and thereby clarify the mechanisms governing early island colonization.

## Materials and methods

### Sample collection

**Fig. 1:**
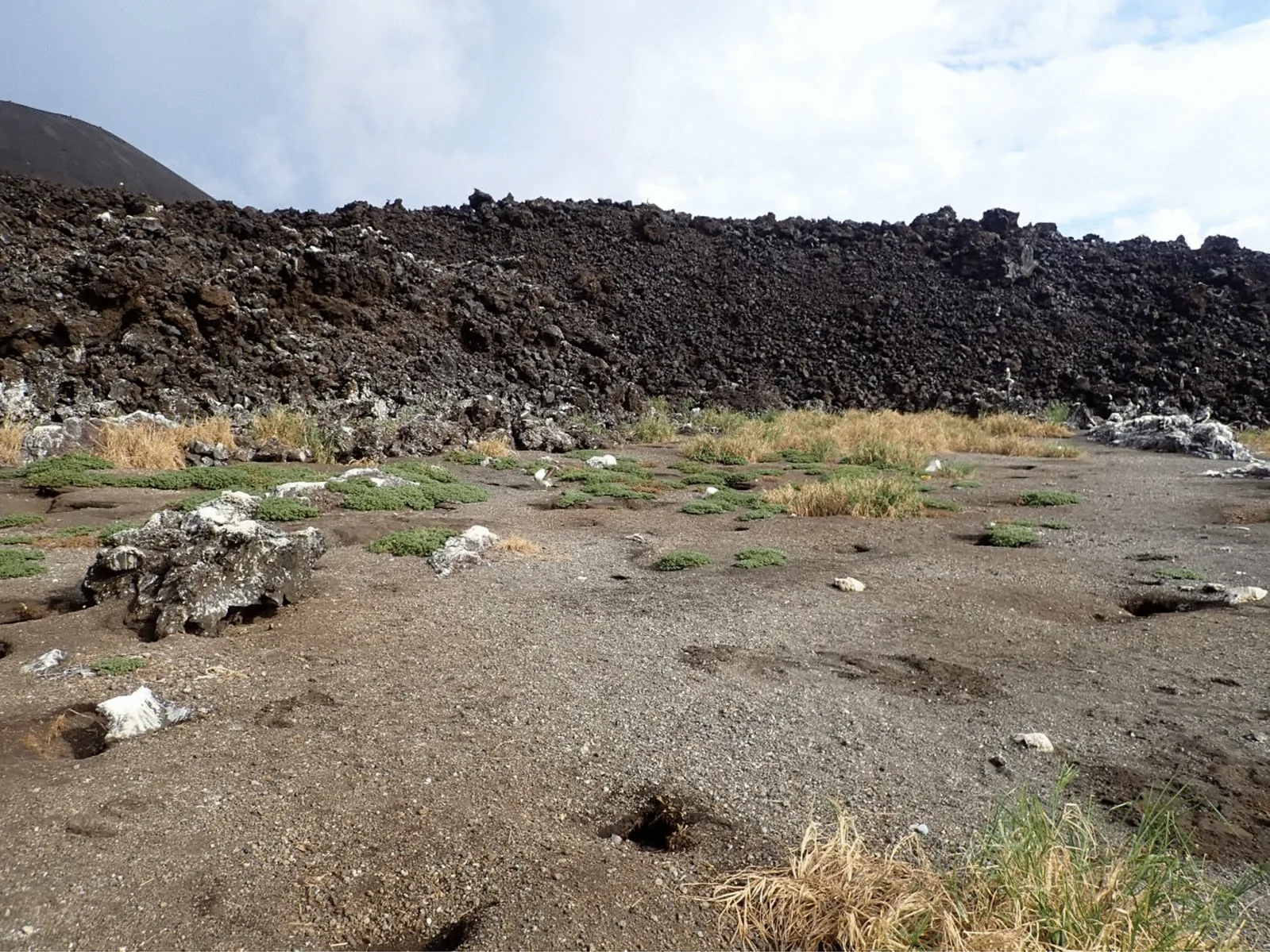
*Eleusine indica* populations in Nishinoshima Island (Date of photography: September 4, 2019)

**Fig. 2:**
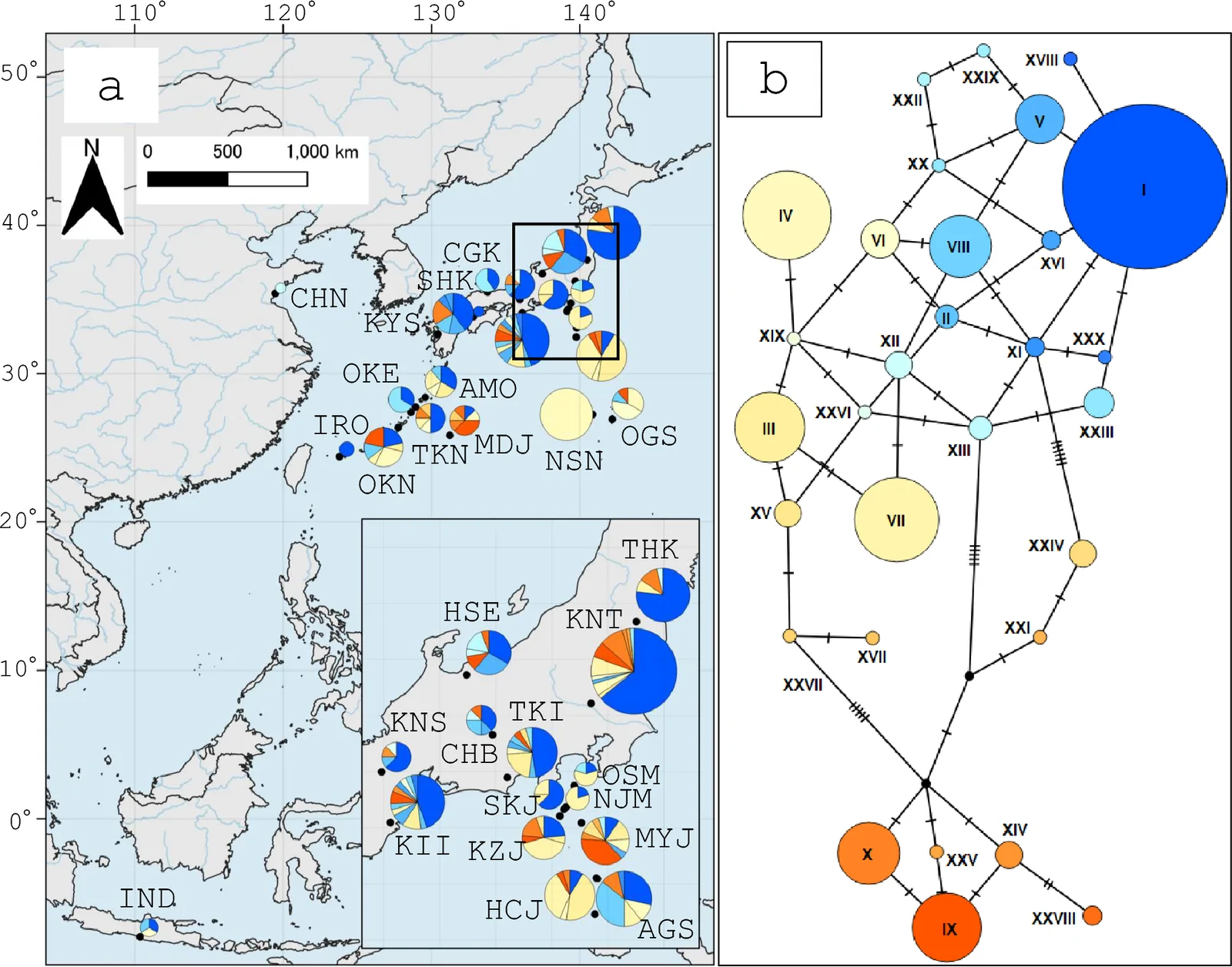
**a**: geographical distribution of cpDNA haplotypes of *E. indica* populations. **b**: chloroplast DNA haplotype network for populations of *E. indica*. The sizes of circles are proportional to the frequencies of haplotypes; the smallest circles represent single individuals. Abbreviations for the populations are provided in Table 2

We collected plant materials from the coastlines of Honshu, Shikoku, Kyushu, and the Ryukyu Islands, as well as from several small islands, including Minami-Daito, Kita-Daito, the Izu Islands, the Ogasawara Islands, and Nishinoshima. Individual samples were collected at intervals of several hundred meters to avoid sampling clones, except on Nishinoshima, where 26 samples were collected from the available distribution area (Fig. 1). Each collected sample was placed in a plastic bag with silica gel and stored in a freezer prior to DNA extraction. The precise location of each sampled individual was determined using a Global Positioning System (GPS; Garmin GPSMAP 62 s). In total, 342 samples were collected from Japan, its surrounding islands, and Indonesia (Fig. 2, Table S1).

### DNA analyses

Total DNA was extracted using a slightly modified CTAB method (Murray and Thompson 1980). To screen for intraspecific polymorphic regions of chloroplast DNA (cpDNA), we attempted to amplify 42 regions using universal cpDNA primers (Chiang et al. 1998; Hamilton 1999; Nishizawa and Watano 2000; Shaw et al. 2005). Five regions, *rpl*32-*trn*L, *trn*V-*ndh*C, *rps*16, *pet*L-*psb*E, and *trn*C-D, exhibited polymorphism within the species; consequently, we conducted sequence analysis for all collected samples using these five regions. PCR was performed using a GeneAmp 9700 cycler (Applied Biosystems). The thermal program consisted of an initial denaturation at 95 °C for 2 min, followed by 35 cycles of denaturation at 95 °C for 30 s, annealing at 50 °C for 30 s, and extension at 72 °C for 90 s, with a final extension at 72 °C for 5 min. The resulting PCR products were purified using Exo-SAP IT (GE Healthcare Limited) and sequenced in both directions using a BigDye Terminator Cycle Sequencing Kit (PE Biosystems). Sequence data were assembled and manually edited using Sequencher 10.4.1 (Gene Codes Corporation).

Multiplexed inter-simple sequence repeat (ISSR) genotyping by sequencing (MIG-seq; Suyama and Matsuki 2015) was performed on the collected individuals. Certain populations with small sample sizes or those from specific areas with sufficient existing data, such as Tokunoshima, Daitojima, and Okinoerabujima, were excluded from this analysis (Table S1). The MIG-seq library was prepared following a slightly modified version of the original protocol. The first PCR utilized the MIG-seq primer set 1 with an annealing temperature of 38 °C, which differed from the original protocol. Subsequently, the first PCR products were diluted and used for the second PCR, which incorporated primer pairs containing tail sequences, Illumina adapter sequences, and dual indices for individual sample identification. The resulting second PCR products, ranging in size from 300 to 800 bp, were multiplexed and sequenced on an Illumina MiSeq platform using a MiSeq Reagent Kit v3.

### Data analyses of cpDNA

Haplotypes were defined as unique combinations of 13 nucleotide variants across the five cpDNA regions (*rpl*32-*trn*L, *trn*V-*ndh*C, *rps*16, *pet*L-*psb*E, and *trn*C-D). Haplotype diversity, nucleotide diversity, and the number of polymorphic sites for each region were calculated using DnaSP v6 (Rozas et al. 2017). FASTA files for each region were concatenated using the concat function in SeqKit v2.9.0 (Shen et al. 2024). Mutational relationships among haplotypes were inferred via the TCS method (Clement et al. 2002) using PopART v1.7 (Leigh and Bryant 2015). Haplotype distribution maps were constructed with QGIS v3.40.5 (QGIS.org 2025). Genetic differentiation (*F*_ST_) between populations was also estimated according to the method of Hudson et al. (1992).

### Data analyses of MIG-seq

Raw sequencing reads were processed by filtering out low-quality reads and short fragments containing adapter sequences using the FASTX Toolkit (http://hannonlab.cshl.edu/fastx_toolkit) and TagDust v1.13 (Lassmann et al. 2009). Subsequently, 80-nucleotide sequences from both ends of the fragments were used for SNP discovery. SNP calling was performed using Stacks v1.47 with the following parameters: a minimum depth of coverage of 3 (-m 3), and a maximum of 2 mismatches allowed between sample loci when building the catalog (-n 2), with all other options set to default. Following stack creation, SNPs for population analysis were filtered to include those with a minor allele frequency (MAF) of at least 0.01 (-min-maf 0.01) and a genotyping rate of at least 80% (-r 0.8) after excluding the individuals with more than 50% missing data using the Populations program in Stacks. To assess Hardy–Weinberg equilibrium (HWE) within each population, a test was conducted using VCFtools v0.1.15 (https://vcftools.github.io/), with* p*-values adjusted according to the Benjamini and Hochberg procedure (1995). SNPs deviating from HWE at adjusted* p*-values < 0.05 in more than one population were excluded from further analysis. Pairwise R2 values for each SNP pair were computed using PLINK v1.90 (Chang et al. 2015). If the value exceeded 0.95, the SNP locus with the lower genotyping rate was eliminated to avoid linkage disequilibrium.

Genetic diversity was assessed by estimating the number of alleles (*N*a), the effective number of alleles (*N*e), observed heterozygosity (*H*o), and expected heterozygosity (*H*e) for each population using GenAlEx v6.5 (Peakall and Smouse 2012). Pairwise genetic differentiation (*F*_ST_) (Wright 1965) was calculated using the program GenAlEx. Based on the values of pairwise *F*_ST_, we performed a principal coordinate analysis (PCoA) using the program GenAlEx. The phylogenetic relationships between individuals were inferred from a maximum likelihood search under the GTR + ASC model using IQ-TREE v.1.6.12 (Nguyen et al. 2015). The phylogenetic tree was visualized using FigTree v.1.4.4. The phylogenetic relationships between populations were inferred from BIONJ (Gascuel 1997) based on the values of pairwise *F*_ST_ in the ape package v.5.8 (Paradis and Schliep 2019) in R. The NJ tree was visualized using the ape package and SplitsTree software version 3 (Huson and Bryant 2006). Population clustering analysis was conducted using the software PopCluster v.1.5.0.0 (Wang 2025) assuming between 1 and 20 ancestral populations (*K*) with 10 independent runs for each *K*. The PopCluster is shown to be more accurate than previous population structure methods even when many populations were assumed, populations are little differentiated and the unbalanced sampling among them (Wang 2025). We applied the level of scaling 4 (very strong) because our dataset includes populations with unbalanced sampling number. We provided the population information of each individual as PopData in an input file. The second order rate of change of the estimated log-likelihood (DLK2) was used to assess the most suitable value of *K*. Spatial genetic structure was assessed by testing the significance of isolation by distance (IBD) using a Mantel test with 9,999 random permutations of the relationship between the matrix of pairwise *F*_ST_/(1 − *F*_ST_) and that of the geographical distance between populations. The geographical distance between populations was calculated using the function distGeo in the geosphere package v.1.5.20. The Mantel test was carried out using the function mantel in the vegan package v.2.7.2.

## Results

### Genetic diversity of cpDNA

The number of haplotypes in the five investigated regions ranged from 2 to 4. Haplotype diversity varied from 0.436 to 0.624, while nucleotide diversity (π) ranged from 0.00033 to 0.00346. Nucleotide polymorphism values ranged from 0.151 to 1.058. Among the five cpDNA regions, the *rpl*32-*trn*L region exhibited the highest levels of polymorphism across all estimated parameters (Table 1).

**Table 1:** Genetic diversity of five regions of cpDNA in *Eleusine indica*

Region	No. of samples analyzed	Length of the region (bp)	No. of haplotype	Haplotype diversity	Nucleotide diversity (π)	Nucleotide polymorphism (θ)	Nucleotide polymorphism θ (per site)
*rpl* 32-*trn*L	419	589	4	0.624	0.00346	1.058	0.00180
*trn*V-*ndh*C	414	742	4	0.597	0.00134	0.303	0.00041
*pet*L-*psb*E	423	749	2	0.436	0.00058	0.151	0.00020
*rp*S16	418	776	2	0.257	0.00033	0.151	0.00019
*trn*C-D	402	728	3	0.561	0.00087	0.304	0.00042

### Haplotype diversity and distribution of cpDNA

Based on the combination of the five polymorphic cpDNA regions, a total of 30 haplotypes were identified in this species (Fig. 2, Table 2). Haplotype diversity tended to be relatively lower in northern parts of Japan, such as the Tohoku and Kanto regions. The number of individuals investigated per population varied from 1 to 67, with an average of 14.4. No haplotype diversity was observed in several populations, including Shikoku, Iriomotejima, China, and Nishinoshima, where the number of samples was 1, 2, 1, and 25, respectively.

**Table 2:** Number of haplotypes identified in each population of* E. indica*

Population	Haplotype															
	Abbreviation	I	II	III	IV	V	VI	VII	VIII	IX	X	XI	XII	XIII	XIV	XV
Tohoku	THK	20						2			3		1			
Kanto	KNT	43		1	3	1	1	5		4	6				1	
Tokai	TKI	11	1	5	1	1			1	1						1
Hokushinnetsu	HSE	6				5				2			1	3		
Chubu	CHB	3				1			2				1			
Kansai	KNS	5	1												1	
Kii Pen	KII	12	1	3		2		1	1	2					1	
Chugoku	CGK	2														
Shikoku	SHK	1														
Kyushu	KYS	6				2			2		3	1				
Ohshima	OSM	1						3								
Niijima	NJM	1						4								
Shikinejima	SKJ	5		1				2								
Kouzushima	KZJ	4		1				7		1	3					1
Miyakejima	MYJ	2		3	2	1				8					1	2
Hachijojima	HCJ	8			3			3	10		3	1				
Aogashima	AGS	2		10	1			8		1	1					
Ogasawara	OGS				3		4		1	1						
Amamioshima	AMO	3		2			1	2	1							
Okinawa	OKN	3		1	4			1	2	3						
Iriomotejima	IRO	2														
Nishinoshima	NSN				25											
Indonesia	IND	1					1		1							
Tokunoshima	TKN	4					1	1			1					
Minamidaitojima	MDJ	1			1					3	1					
Okinoerabujima	OKE	2														
China	CHN												1			
Total		148	3	27	43	13	8	39	21	26	21	2	4	3	4	4
Proportion(%)		0.379	0.008	0.069	0.110	0.033	0.021	0.100	0.054	0.067	0.054	0.005	0.010	0.008	0.010	0.010

Population	Haplotype															
	Abbreviation	XVI	XVII	XVIII	XIX	XX	XXI	XXII	XXIII	XXIV	XXV	XXVI	XXVII	XXVIII	XXIV	XXX
Tohoku	THK															
Kanto	KNT										1				1	
Tokai	TKI								1							
Hokushinnetsu	HSE													1		
Chubu	CHB													1		
Kansai	KNS											1				
Kii Pen	KII	1			1	1										1
Chugoku	CGK									3						
Shikoku	SHK															
Kyushu	KYS	1														
Ohshima	OSM									1						
Niijima	NJM															
Shikinejima	SKJ															
Kouzushima	KZJ															
Miyakejima	MYJ						1	1								
Hachijojima	HCJ															
Aogashima	AGS															
Ogasawara	OGS															
Amamioshima	AMO															
Okinawa	OKN															
Iriomotejima	IRO															
Nishinoshima	NSN															
Indonesia	IND															
Tokunoshima	TKN												1			
Minamidaitojima	MDJ		1	1												
Okinoerabujima	OKE								4							
China	CHN															
Total		2	1	1	1	1	1	1	5	4	1	1	1	2	1	1
Proportion(%)		0.005	0.003	0.003	0.003	0.003	0.003	0.003	0.013	0.010	0.003	0.003	0.003	0.005	0.003	0.003

Haplotype I was the most dominant, accounting for 38.0% of all investigated individuals and showing a wide distribution across Japan (Fig. 2, Table 2). Other frequent haplotypes included Haplotype IV (11.0%), Haplotype VII (10.0%), Haplotype III (6.9%), and Haplotype IX (6.7%). Eleven haplotypes were identified as singletons (occurring in only one individual). No clear geographical trend was observed in the overall distribution of cpDNA haplotypes. The overall genetic differentiation between populations was estimated at *F*_ST_ = 0.309.

### Genetic diversity of nuclear SNPs

The genetic diversity of 337 *E. indica* individuals was characterized using 964 SNP markers generated via the MIG-seq platform. The number of investigated individuals per population ranged from 2 to 68, with an average of 14.6 (Table 3). The effective number of alleles (*N*e) ranged from 1.005 to 1.368. Observed heterozygosity (*H*o) varied from 0.004 to 0.039, while unbiased expected heterozygosity (u*H*e) ranged from 0.003 to 0.228. Notably, the Nishinoshima population exhibited the lowest values for u*H*e. The inbreeding coefficient (*F*_IS_) for all populations showed high positive values, with the exception of the Nishinoshima population.

**Table 3:** Population genetic parameters using 964 SNPs data from MIG-seq analysis

Population	Abbreviation	N	*N*a	*N*e	*H*o	*H*e	u*H*e	*F*_IS_
Tohoku	THK	29	1.721	1.321	0.010	0.198	0.201	0.946
Kanto	KNT	68	1.794	1.338	0.014	0.209	0.211	0.907
Tokai	TKI	38	1.793	1.338	0.009	0.210	0.213	0.952
Hokushinnetsu	HSE	15	1.580	1.250	0.012	0.161	0.167	0.904
Chubu	CHB	8	1.523	1.304	0.006	0.183	0.195	0.956
Kansai	KNS	7	1.463	1.285	0.039	0.169	0.183	0.745
Kii Pen	KII	11	1.657	1.319	0.007	0.199	0.209	0.956
Chugoku	CGK	5	1.356	1.318	0.016	0.168	0.188	0.882
Kyushu	KYS	13	1.567	1.298	0.006	0.181	0.189	0.949
Ohshima	OSM	5	1.560	1.337	0.016	0.204	0.228	0.890
Niijima	NJM	5	1.317	1.228	0.007	0.129	0.144	0.945
Shikinejima	SKJ	8	1.463	1.301	0.008	0.174	0.186	0.938
Kouzushima	KZJ	17	1.486	1.235	0.009	0.145	0.150	0.926
Miyakejima	MYJ	21	1.710	1.304	0.009	0.189	0.194	0.923
Hachijojima	HCJ	27	1.702	1.368	0.006	0.220	0.224	0.947
Aogashima	AGS	20	1.533	1.211	0.015	0.140	0.144	0.869
Ogasawara	OGS	7	1.372	1.216	0.006	0.131	0.142	0.948
Okinawa	OKN	5	1.198	1.153	0.004	0.086	0.096	0.941
Iriomotejima	IRO	2	1.216	1.199	0.024	0.105	0.140	0.715
Nishinoshima	NSN	25	1.029	1.005	0.004	0.003	0.003	− 0.019
Indoneshia	IND	3	1.246	1.194	0.007	0.110	0.132	0.924
Mean		16.1	1.425	1.227	0.010	0.138	0.148	0.910

### Genetic structure of nuclear SNPs

**Fig. 3:**
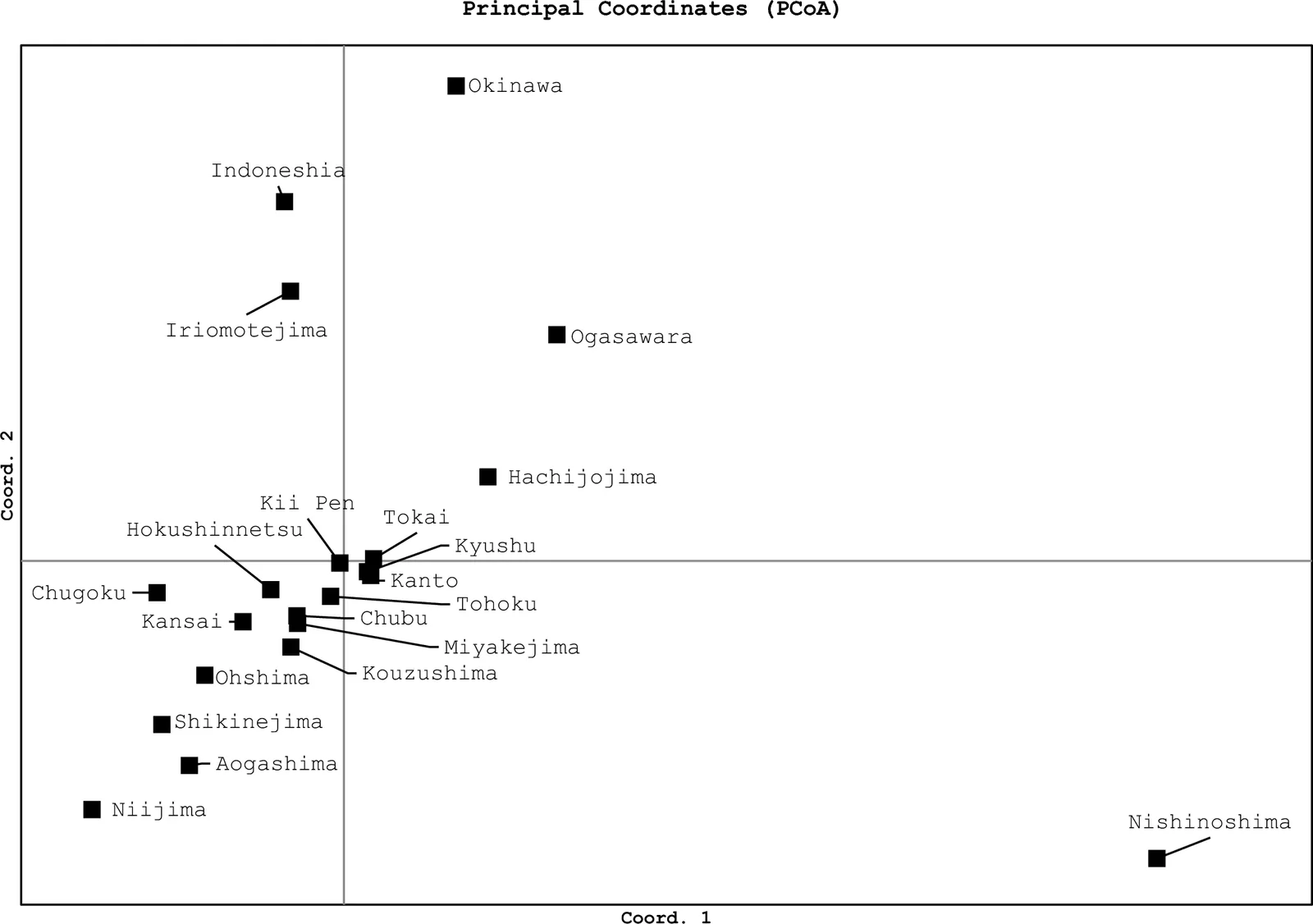
A principal coordinate analysis (PCoA) plot based on pairwise *F*_ST_ at population level

**Fig. 4:**
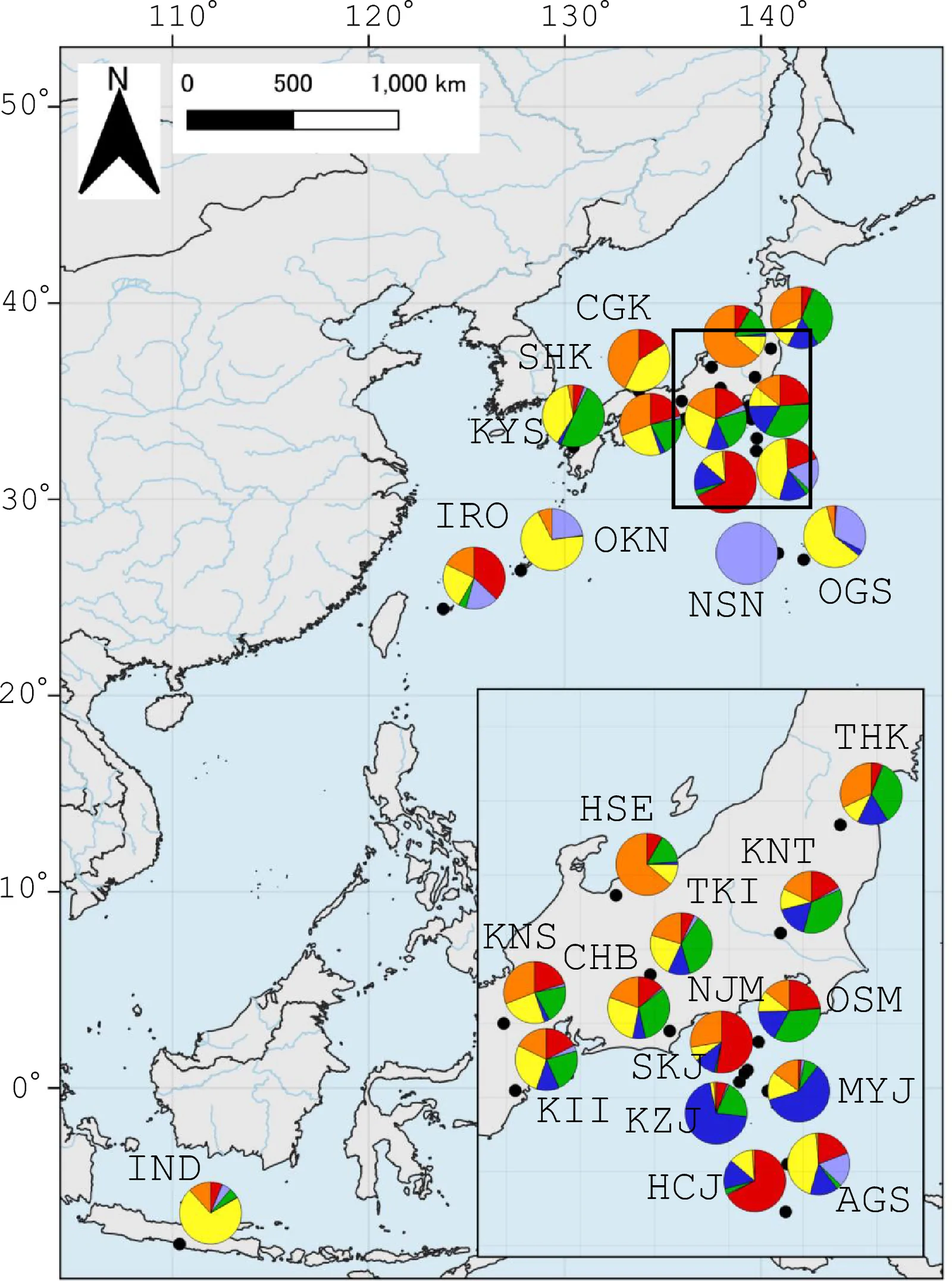
The result of PopCluster analysis with *K* = 5 in *E. indica*. Abbreviations for the populations are provided in Table 2

**Fig. 5:**
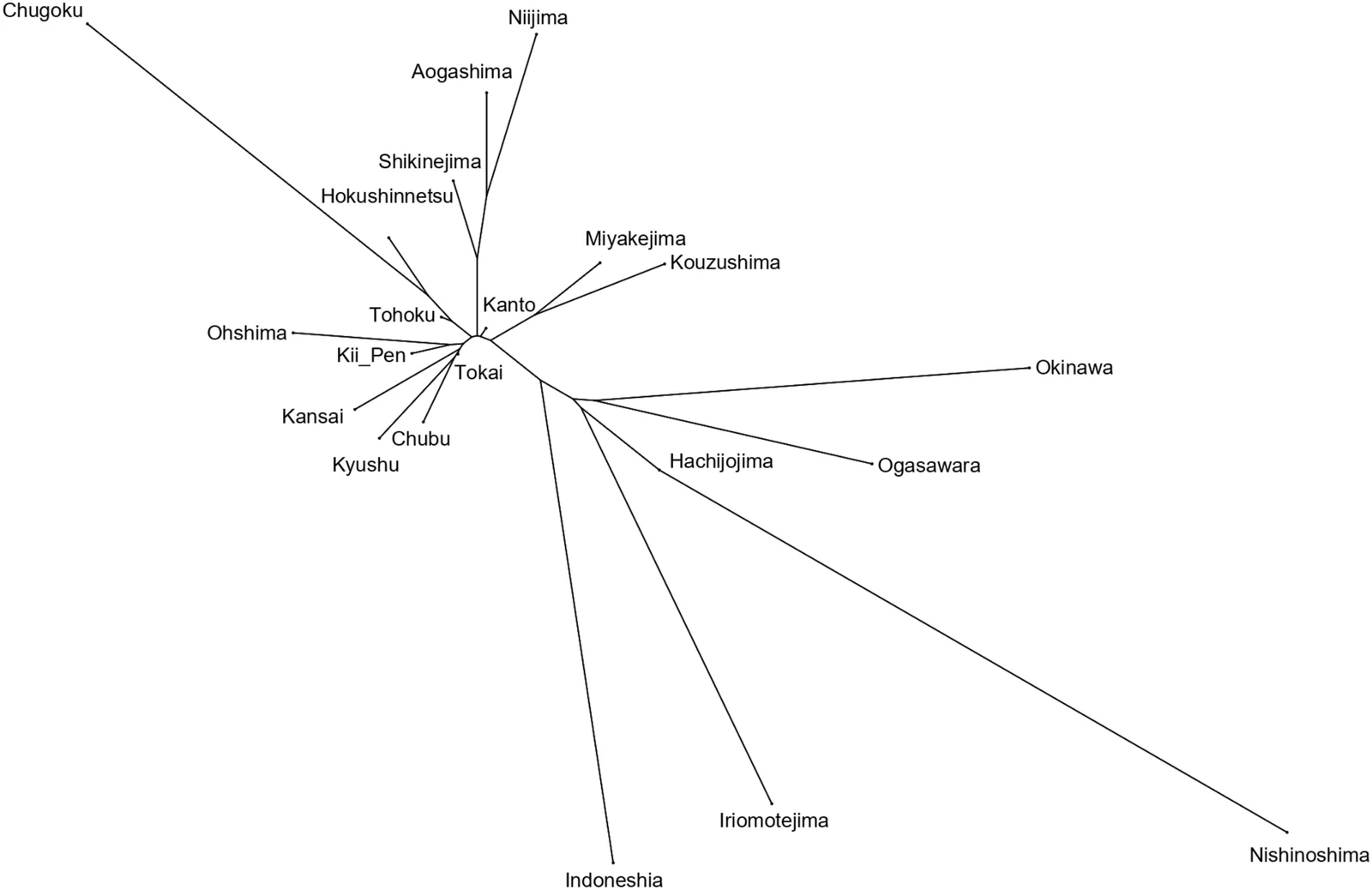
The phylogenetic tree at population level in *E. indica* based on pairwise *F*_ST_ at population level

**Fig. 6:**
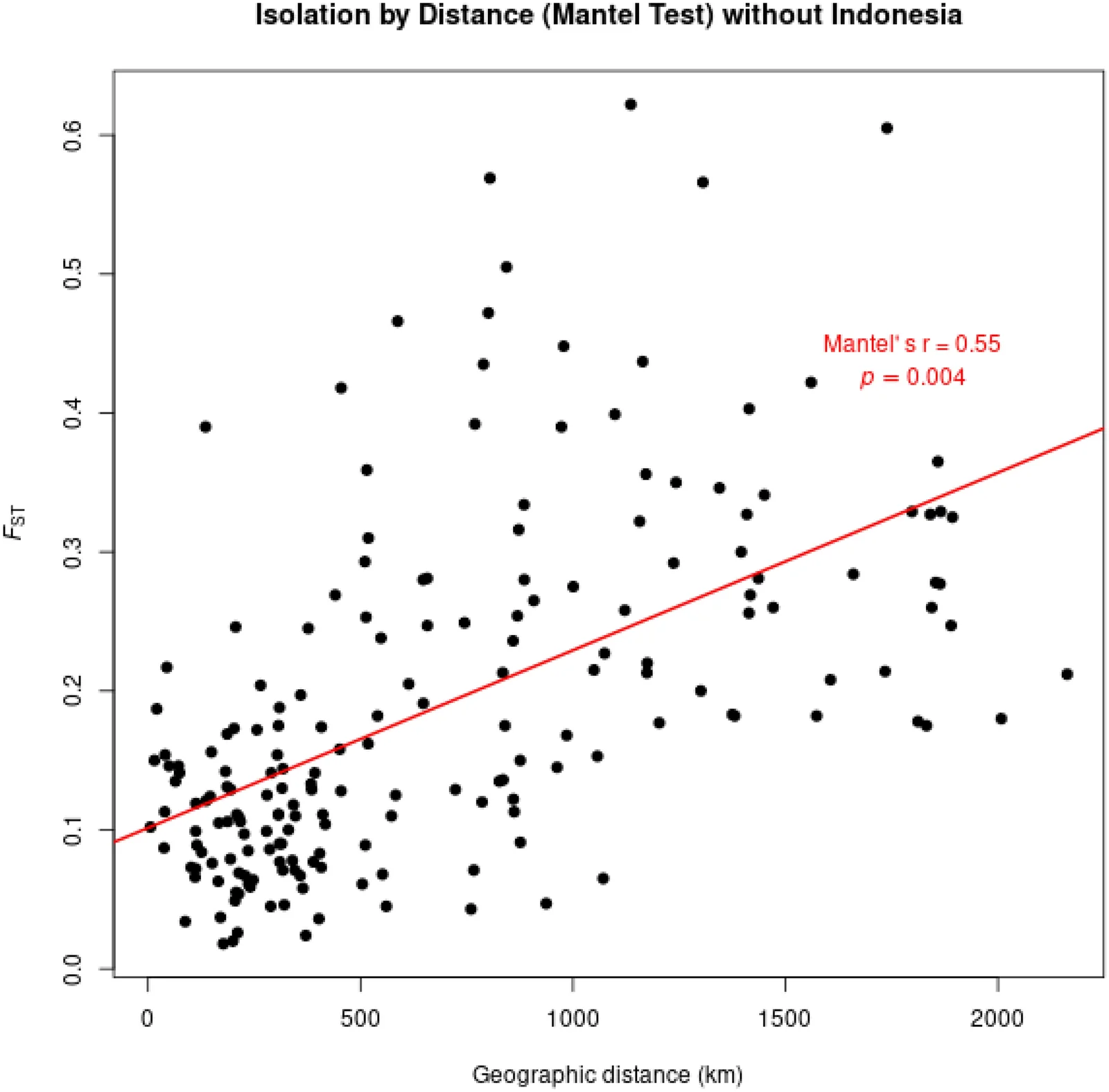
Isolation by distance (IBD) within Japanese populations by Mantel test (r = 0.55, *p*  =  0.006)

Pairwise genetic differentiation (*F*_ST_) among populations ranged from 0.020 to 0.622, with an average of 0.182 (Table S2). PCoA based on pairwise *F*_ST_ revealed that the Nishinoshima population was significantly isolated from other populations, particularly along the first and second principal coordinate axes, which explained 19.6% and 13.50% of the total variance, respectively (Fig. 3). Additionally, populations from Okinawa, Indonesia, and the Ogasawara Islands showed some separation from the main population group along the second axis. Population cluster analysis (PopCluster) indicated that the most likely number of genetic clusters was *K* = 6 (Fig. S1). Although no clear broad-scale geographical trend was observed, the "green" cluster predominated in Honshu, Shikoku, and Kyushu, whereas the "light-blue" cluster was relatively more frequent in Iriomotejima, Okinawa, Aogashima, the Ogasawara Islands, and Nishinoshima (Fig. 4, Fig. S2). Population-level phylogenetic analysis revealed that the Nishinoshima population is most closely related to the Ogasawara population, followed by Okinawa and Indonesia (Fig. 5). In contrast, populations in mainland Japan (Honshu, Shikoku, and Kyushu) exhibited no distinct geographical structuring. The individual-level phylogenetic tree showed a topology highly consistent with the population-level tree (Fig. S3). We also assessed the correlation between pairwise *F*_ST_ and geographical distances to test for isolation-by-distance (IBD) within Japanese populations (Fig. 6). A highly significant positive correlation was observed (*r* = 0.55, Mantel test, *p* < 0.004), indicating that IBD is a prominent feature of the genetic structure of this species in mainland Japan. This association remained significant even when the Indonesian population was included in the analysis (*r* = 0.43, Mantel test, *p* < 0.049, Fig. S4).

## Discussions

### Genetic diversity and genetic structure of* E. indica* populations

The genetic diversity of chloroplast DNA (cpDNA) in *E. indica* is relatively moderate compared to other species (Aoki et al. 2016; San Jose-Maldia et al. 2017; Widmer and Baltisberge 1999). We identified 30 haplotypes across five cpDNA regions; however, no distinct geographical trend was observed. Generally, genetic differentiation of cpDNA among populations tends to be high due to its maternal inheritance; however, in this species, the differentiation was relatively low (*F*_ST_ = 0.309), suggesting the occurrence of long-distance gene flow mediated by seed dispersal.

Similarly, nuclear SNP analysis revealed relatively moderate genetic diversity compared to other species (Kusuma et al. 2022; Nomura et al. 2025; Onosato et al. 2021; Shiraishi et al. 2025; Utomo et al. 2025). Despite its wide distribution, genetic differentiation among populations was low (*F*_ST_ = 0.176), and the observed pattern of isolation-by-distance (IBD) indicates that while long-distance gene flow occurs, it is constrained by geographical distance. Given that *E. indica* is a cosmopolitan species, frequent long-distance seed dispersal likely maintains this genetic connectivity. The difference in *F*_ST_ between chloroplast and nuclear DNA is primarily due to their different modes of inheritance. Chloroplast DNA is maternally inherited and relies mainly on seed dispersal, whereas nuclear DNA is biparentally inherited and is dispersed via both pollen and seeds. Consequently, chloroplast DNA also exhibits a higher *F*_ST_ value in this study.

The seeds of *E. indica* are remarkably small (1–2 mm), facilitating transport via strong winds, ocean currents, or seabirds (Aoyama et al. 2012; Nathan et al. 2002). Known for long-distance seed dispersal via ocean currents, mangrove species, *Rhizophora mucronata* had the average *F*_ST_ value of 0.625 in southeast Asia (nuclear microsatellites; Wee et al. 2014), and *Ipomoea pes-caprae* had the average *F*_ST_ value of 0.472 within the western Pacific (nuclear EST sequences; Miryeganeh et al. 2014). *Hibiscus tiliaceus* had much lower *F*_ST_ values, 0.254 between the Atlantic and Pacific populations and 0.289 between the Atlantic and Indian Ocean populations (cpDNA PCR-SSCP and PCR-SSP; Takayama et al. 2006). Such seed dispersal is relied on ocean currents, and thus there is certain direction. The difference of *F*_ST_ between species might be number of seeds production for every year, for example, mangrove species produce a limited number of large seeds, but *Ipomoea* species produce large number of small seeds, and *Hibiscus* species produce much large number of tiny seeds. Germination of seed in *E. indica* was greater in higher temperature and was tolerant of salt stress (Chauhan and Johnson 2008). Therefore, the survival rate of this species might be higher in tropical and subtropical islands.

### Genetic diversity of* E. indica* in Nishinoshima Island

Our results showed that all 25 individuals sampled from Nishinoshima shared a single cpDNA haplotype (Haplotype IV). This extreme genetic uniformity strongly suggests a pronounced founder effect, indicating that the current population likely originated from a very small number of seeds, potentially even a single dispersal event. Nuclear DNA analysis further confirmed this lack of diversity, with exceptionally low observed and expected heterozygosity (0.004 and 0.003, respectively). While the inbreeding coefficient (*F*_IS_) was − 0.019, which might suggest a random mating system, this estimate is likely confounded by the extremely low genetic diversity of the population. Given that we collected 25 individuals of *E. indica* from a coastal survival site of roughly 200 m^2^, the only such area remaining on the island, it is highly probable that the initial population originated from a single individual. It is thought that subsequent self-fertilization maintained the population until recently. Unfortunately, this area has since been buried by volcanic ash and lava from the 2020 eruption (Mori et al. 2020); consequently, further monitoring of the population is no longer possible.

### Origin of the* E. indica* in Nishinoshima Island

The Nishinoshima population is genetically most similar to the Ogasawara population, followed by those in Okinawa, Indonesia, the Izu Islands, and Iriomotejima (Fig. 5, Fig. S3). These phylogenetic trees suggest a migration route for this species likely originating in Indonesia and reaching Nishinoshima via Okinawa and Ogasawara. The cpDNA haplotype of the Nishinoshima population is fixed to Haplotype IV, which is relatively dominant in Ogasawara, the Izu Islands, and the Ryukyu Islands. Although this haplotype has not been identified in the Indonesian population, this may be due to the limited sample size investigated in that region.

Seabirds such as *Sula leucogaster* and *Ardenna pacifica* may contribute to this long-distance gene flow (Aoyama et al. 2012). There are also records of *E. indica* seeds attached to seabird feathers in the Ogasawara Islands (Ono and Sugawara 1981), leading to the hypothesis that *E. indica* on Nishinoshima was transported from distant islands by seabirds. *Sula leucogaster*, the most abundant species confirmed to inhabit Nishinoshima (Kawakami and Oyamada 2020), prefers subtropical and tropical habitats. These birds undertake long-distance migrations, with reported movements from the Ogasawara Islands to New Guinea (Yamashina Institute for Ornithology 2002) and from Okinawa to New Guinea via the Philippines (Kohno et al. 1997). Such long-distance migrations of *S. leucogaster* likely facilitate the dispersal of *E. indica* seeds from tropical regions to Nishinoshima via Okinawa and Ogasawara. The seeds of *E. indica* are characterized by their minute size (typically 1.0–1.5 mm), allowing them to easily lodge in small crevices such as feathers or the muddy feet of seabirds. Furthermore, these seeds have been observed to develop a slight mucilaginous or adhesive quality when moistened, significantly enhancing their capacity for epizooichory (Ridley 1930; Shimada and Ellner 1983). This physical adaptation supports the hypothesis that seabirds act as primary vectors for the long-distance dispersal of this species to remote volcanic islands like Nishinoshima, where seeds are often deposited near nesting colonies.

In the first survey of Nishinoshima in 1969, three plant species were recorded: *E. indica*, *E. crus-galli*, and *P. oleracea*. Given the stringent landing restrictions imposed on Nishinoshima, the possibility of anthropogenic introduction is extremely low. A molecular investigation of *P. oleracea* on Nishinoshima concluded that the population was genetically closest to the Chichijima (Ogasawara) population, located approximately 130 km away (Noda et al. 2025). While our findings are consistent with this report, our results further elucidate the potential migration route of *E. indica* from tropical areas. These results provide important insights into the processes of early ecosystem establishment.

## Supplementary Information

Below is the link to the electronic supplementary material.

Supplementary Material 1

## Data Availability

Raw GBS data are available on DDBJ's Sequence Read Archive (DRA) as ‘BioProject’ PRJDB40141 and ‘BioSamples’ SAMD01797291-SAMD01797311 (SSUB045627).
