# Supplementary material for: Origin of the Eleusine indica population on the active volcanic Nishinoshima Island, Japan
Source: J Plant Res. 2026 Aug 7;139(5):717–30. doi: 10.1007/s10265-026-01738-9 (PMC13588880; doi:10.1007/s10265-026-01738-9)
Supplement: Supplementary file 1 [file 10265_2026_1738_MOESM1_ESM.pdf]

Supplemental file

Title: Origin of the *Eleusine indica* population on the active volcanic Nishinoshima Island, Japan

Authors: Koutaro Maeda[1], Takaki Aihara[2], Kentaro Uchiyama[3], Mengying Cai[1], Widiyatno[4], Takashi Kamijo[2] and Yoshihiko Tsumura[2,5]

1: Graduate School of Life and Environmental Sciences, University of Tsukuba, Tsukuba, Ibaraki 305-8572, Japan

2: Institute of Life and Environmental Sciences, University of Tsukuba, Tsukuba, Ibaraki 305-8572, Japan

3: Department of Forest Molecular Genetics and Biotechnology, Forestry and Forest Products Research Institute, Tsukuba, Ibaraki, 305-8687, Japan

4: Faculty of Forestry, Gadjah Mada University, Bulaksumur, Yogyakarta 55281, Indonesia

5: Author for correspondence: Yoshihiko Tsumura, phone: +81-29-853-4629, E-mail: tsumura.yoshihiko.ke@u.tsukuba.ac.jp

Table S1. Collected samples in *Eleusine indica*

Population	Prefecture	Latitude	Longitude
Tohoku	Miyagi	38.270	140.761
Tohoku	Miyagi	38.158	140.888
Tohoku	Miyagi	38.118	140.716
Tohoku	Miyagi	38.082	140.876
Tohoku	Miyagi	38.069	140.661
Tohoku	Miyagi	38.044	140.905
Tohoku	Miyagi	38.024	140.626
Tohoku	Miyagi	37.930	140.604
Tohoku	Miyagi	37.873	140.546
Tohoku	Miyagi	38.274	140.866
Tohoku	Fukushima	37.821	140.507
Tohoku	Fukushima	37.728	140.461
Tohoku	Fukushima	37.659	140.493
Tohoku	Fukushima	37.584	140.446
Tohoku	Fukushima	37.542	139.973
Tohoku	Fukushima	37.539	140.398
Tohoku	Fukushima	37.537	140.397
Tohoku	Fukushima	37.537	140.108
Tohoku	Fukushima	37.518	139.918
Tohoku	Fukushima	37.492	140.206
Tohoku	Fukushima	37.480	140.349
Tohoku	Fukushima	37.287	140.377
Tohoku	Fukushima	37.213	140.327
Tohoku	Fukushima	37.152	140.250
Tohoku	Fukushima	37.122	140.178
Tohoku	Fukushima	37.104	140.862
Tohoku	Fukushima	37.066	140.939
Kanto	Tochigi	37.090	140.132
Kanto	Tochigi	37.054	140.112
Kanto	Tochigi	36.985	140.070

Kanto	Tochigi	36.931	140.027
Kanto	Tochigi	36.930	140.008
Kanto	Tochigi	36.886	139.971
Kanto	Tochigi	36.834	139.950
Kanto	Tochigi	36.798	139.911
Kanto	Tochigi	36.780	139.713
Kanto	Tochigi	36.774	139.816
Kanto	Tochigi	36.762	139.719
Kanto	Tochigi	36.757	139.606
Kanto	Tochigi	36.744	139.656
Kanto	Tochigi	36.728	139.671
Kanto	Tochigi	36.639	139.727
Kanto	Tochigi	36.598	139.788
Kanto	Tochigi	36.548	139.930
Kanto	Tochigi	36.442	140.002
Kanto	Tochigi	36.431	140.072
Kanto	Tochigi	36.309	139.761
Kanto	Tochigi	36.288	139.560
Kanto	Gunma	36.778	138.945
Kanto	Gunma	36.622	138.601
Kanto	Gunma	36.608	138.638
Kanto	Gunma	36.602	139.064
Kanto	Gunma	36.548	138.953
Kanto	Gunma	36.450	139.275
Kanto	Gunma	36.450	139.275
Kanto	Gunma	36.450	139.275
Kanto	Gunma	36.450	139.275
Kanto	Gunma	36.450	139.275
Kanto	Gunma	36.432	139.015
Kanto	Gunma	36.389	139.275
Kanto	Gunma	36.368	139.161
Kanto	Gunma	36.265	139.296

Kanto	Gunma	36.265	139.296
Kanto	Gunma	36.265	139.296
Kanto	Gunma	36.265	139.296
Kanto	Ibaraki	36.868	140.742
Kanto	Ibaraki	36.758	140.692
Kanto	Ibaraki	36.603	140.636
Kanto	Ibaraki	36.485	140.551
Kanto	Ibaraki	36.362	140.118
Kanto	Ibaraki	36.309	140.339
Kanto	Ibaraki	36.286	139.877
Kanto	Ibaraki	36.235	140.296
Kanto	Ibaraki	36.218	140.121
Kanto	Ibaraki	36.213	140.100
Kanto	Ibaraki	36.207	140.074
Kanto	Ibaraki	36.138	140.081
Kanto	Ibaraki	36.109	140.120
Kanto	Ibaraki	36.109	140.120
Kanto	Ibaraki	36.109	140.120
Kanto	Ibaraki	36.097	140.086
Kanto	Saitama	36.256	139.119
Kanto	Saitama	36.175	139.193
Kanto	Saitama	36.037	139.459
Kanto	Saitama	36.037	139.459
Kanto	Saitama	36.037	139.459
Kanto	Saitama	36.037	139.459
Kanto	Saitama	35.986	139.384
Kanto	Saitama	35.899	139.467
Kanto	Saitama	35.899	139.467
Kanto	Saitama	35.899	139.467
Kanto	Saitama	35.899	139.467
Kanto	Chiba	35.759	140.107
Kanto	Chiba	35.649	140.123

Kanto	Chiba	35.573	140.379
Kanto	Chiba	35.473	140.412
Kanto	Chiba	35.463	139.876
Kanto	Chiba	35.281	139.926
Kanto	Chiba	35.009	139.976
Kanto	Chiba	34.923	139.830
Kanto	Chiba	34.904	139.888
Kanto	Kanagawa	35.467	139.625
Kanto	Kanagawa	35.431	139.402
Kanto	Kanagawa	35.375	139.206
Kanto	Kanagawa	35.319	139.551
Kanto	Kanagawa	35.288	139.173
Kanto	Kanagawa	35.200	139.138
Kanto	Kanagawa	35.146	139.114
Ohshima	Tokyo	34.780	139.371
Ohshima	Tokyo	34.763	139.430
Ohshima	Tokyo	34.761	139.434
Ohshima	Tokyo	34.747	139.365
Ohshima	Tokyo	34.690	139.400
Niijima	Tokyo	34.377	139.257
Niijima	Tokyo	34.372	139.259
Niijima	Tokyo	34.369	139.248
Niijima	Tokyo	34.363	139.253
Niijima	Tokyo	34.362	139.246
Shikinejima	Tokyo	34.336	139.212
Shikinejima	Tokyo	34.334	139.215
Shikinejima	Tokyo	34.331	139.209
Shikinejima	Tokyo	34.331	139.215
Shikinejima	Tokyo	34.327	139.208
Shikinejima	Tokyo	34.326	139.225
Shikinejima	Tokyo	34.325	139.221
Shikinejima	Tokyo	34.319	139.215

Kouzujima	Tokyo	34.218	139.138
Kouzujima	Tokyo	34.214	139.139
Kouzujima	Tokyo	34.214	139.144
Kouzujima	Tokyo	34.213	139.141
Kouzujima	Tokyo	34.212	139.145
Kouzujima	Tokyo	34.203	139.152
Kouzujima	Tokyo	34.197	139.152
Kouzujima	Tokyo	34.191	139.134
Miyakejima	Tokyo	34.121	139.527
Miyakejima	Tokyo	34.120	139.536
Miyakejima	Tokyo	34.119	139.512
Miyakejima	Tokyo	34.119	139.504
Miyakejima	Tokyo	34.114	139.552
Miyakejima	Tokyo	34.111	139.556
Miyakejima	Tokyo	34.108	139.558
Miyakejima	Tokyo	34.106	139.495
Miyakejima	Tokyo	34.101	139.492
Miyakejima	Tokyo	34.095	139.561
Miyakejima	Tokyo	34.086	139.561
Miyakejima	Tokyo	34.083	139.560
Miyakejima	Tokyo	34.076	139.478
Miyakejima	Tokyo	34.075	139.481
Miyakejima	Tokyo	34.075	139.558
Miyakejima	Tokyo	34.072	139.480
Miyakejima	Tokyo	34.068	139.482
Miyakejima	Tokyo	34.064	139.551
Miyakejima	Tokyo	34.060	139.544
Miyakejima	Tokyo	34.057	139.538
Miyakejima	Tokyo	34.050	139.525
Miyakejima	Tokyo	34.049	139.511
Hachijoujima	Tokyo	33.154	139.749
Hachijoujima	Tokyo	33.142	139.756

Hachijoujima	Tokyo	33.132	139.767
Hachijoujima	Tokyo	33.131	139.798
Hachijoujima	Tokyo	33.131	139.719
Hachijoujima	Tokyo	33.121	139.796
Hachijoujima	Tokyo	33.121	139.814
Hachijoujima	Tokyo	33.121	139.800
Hachijoujima	Tokyo	33.116	139.753
Hachijoujima	Tokyo	33.116	139.793
Hachijoujima	Tokyo	33.113	139.780
Hachijoujima	Tokyo	33.113	139.781
Hachijoujima	Tokyo	33.111	139.773
Hachijoujima	Tokyo	33.104	139.793
Hachijoujima	Tokyo	33.104	139.773
Hachijoujima	Tokyo	33.102	139.774
Hachijoujima	Tokyo	33.098	139.782
Hachijoujima	Tokyo	33.096	139.846
Hachijoujima	Tokyo	33.080	139.827
Hachijoujima	Tokyo	33.079	139.841
Hachijoujima	Tokyo	33.076	139.785
Hachijoujima	Tokyo	33.076	139.841
Hachijoujima	Tokyo	33.073	139.816
Hachijoujima	Tokyo	33.073	139.816
Hachijoujima	Tokyo	33.072	139.798
Hachijoujima	Tokyo	33.071	139.803
Hachijoujima	Tokyo	33.068	139.814
Hachijoujima	Tokyo	33.066	139.809
Aogashima	Tokyo	32.467	139.765
Aogashima	Tokyo	32.466	139.762
Aogashima	Tokyo	32.464	139.767
Aogashima	Tokyo	32.463	139.764
Aogashima	Tokyo	32.463	139.759
Aogashima	Tokyo	32.462	139.761

Aogashima	Tokyo	32.461	139.772
Aogashima	Tokyo	32.459	139.769
Aogashima	Tokyo	32.459	139.768
Aogashima	Tokyo	32.459	139.765
Aogashima	Tokyo	32.458	139.764
Aogashima	Tokyo	32.458	139.763
Aogashima	Tokyo	32.456	139.773
Aogashima	Tokyo	32.456	139.773
Aogashima	Tokyo	32.455	139.763
Aogashima	Tokyo	32.455	139.772
Aogashima	Tokyo	32.454	139.774
Aogashima	Tokyo	32.453	139.772
Aogashima	Tokyo	32.453	139.763
Aogashima	Tokyo	32.452	139.763
Aogashima	Tokyo	32.452	139.771
Aogashima	Tokyo	32.451	139.770
Aogashima	Tokyo	32.450	139.774
Aogashima	Tokyo	32.449	139.769
Nishinoshima	Tokyo	27.246	140.873
Nishinoshima	Tokyo	27.246	140.873
Nishinoshima	Tokyo	27.246	140.873
Nishinoshima	Tokyo	27.246	140.873
Nishinoshima	Tokyo	27.246	140.873
Nishinoshima	Tokyo	27.246	140.873
Nishinoshima	Tokyo	27.246	140.873
Nishinoshima	Tokyo	27.246	140.873
Nishinoshima	Tokyo	27.246	140.873
Nishinoshima	Tokyo	27.246	140.873
Nishinoshima	Tokyo	27.246	140.873
Nishinoshima	Tokyo	27.246	140.873
Nishinoshima	Tokyo	27.246	140.873
Nishinoshima	Tokyo	27.246	140.873

Nishinoshima	Tokyo	27.246	140.873
Nishinoshima	Tokyo	27.246	140.873
Nishinoshima	Tokyo	27.246	140.873
Nishinoshima	Tokyo	27.246	140.873
Nishinoshima	Tokyo	27.246	140.873
Nishinoshima	Tokyo	27.246	140.873
Nishinoshima	Tokyo	27.246	140.873
Nishinoshima	Tokyo	27.246	140.873
Nishinoshima	Tokyo	27.246	140.873
Nishinoshima	Tokyo	27.246	140.874
Nishinoshima	Tokyo	27.246	140.873
Nishinoshima	Tokyo	27.246	140.874
Nishinoshima	Tokyo	27.246	140.873
Nishinoshima	Tokyo	27.246	140.873
Ogasawara	Tokyo	27.095	142.195
Ogasawara	Tokyo	27.095	142.193
Ogasawara	Tokyo	27.095	142.196
Ogasawara	Tokyo	27.074	142.208
Ogasawara	Tokyo	27.073	142.201
Ogasawara	Tokyo	27.073	142.204
Ogasawara	Tokyo	27.073	142.204
Ogasawara	Tokyo	27.069	142.215
Ogasawara	Tokyo	26.640	142.161
Ogasawara	Tokyo	26.639	142.159
Ogasawara	Tokyo	26.639	142.158
Ogasawara	Tokyo	26.456	142.160
Tokai	Shizuoka	35.129	138.938
Tokai	Shizuoka	35.058	139.070
Tokai	Shizuoka	35.026	138.925
Tokai	Shizuoka	35.023	138.787
Tokai	Shizuoka	35.022	138.898
Tokai	Shizuoka	34.985	139.094

Tokai	Shizuoka	34.923	138.931
Tokai	Shizuoka	34.883	139.109
Tokai	Shizuoka	34.856	138.923
Tokai	Shizuoka	34.844	139.078
Tokai	Shizuoka	34.841	137.750
Tokai	Shizuoka	34.746	139.000
Tokai	Shizuoka	34.675	138.951
Tokai	Shizuoka	34.671	137.707
Tokai	Shizuoka	34.640	138.111
Tokai	Shizuoka	34.635	138.890
Tokai	Shizuoka	34.619	138.823
Tokai	Yamanashi	35.846	138.369
Tokai	Yamanashi	35.646	138.714
Tokai	Yamanashi	35.628	139.047
Tokai	Gifu	35.429	136.843
Tokai	Gifu	35.395	136.693
Tokai	Aichi	35.204	136.868
Tokai	Aichi	35.123	136.907
Tokai	Aichi	35.040	137.048
Tokai	Aichi	35.035	137.513
Tokai	Aichi	34.987	137.087
Tokai	Aichi	34.939	137.167
Tokai	Aichi	34.926	137.513
Tokai	Aichi	34.861	137.177
Tokai	Aichi	34.584	137.020
Hokushinetsu	Niigata	37.426	138.645
Hokushinetsu	Niigata	37.338	138.475
Hokushinetsu	Niigata	37.326	138.783
Hokushinetsu	Niigata	37.271	138.838
Hokushinetsu	Niigata	37.168	138.112
Hokushinetsu	Niigata	37.164	138.952
Hokushinetsu	Niigata	37.034	137.869

Hokushinetsu	Niigata	36.980	138.813
Hokushinetsu	Ishikawa	36.535	136.528
Hokushinetsu	Ishikawa	36.354	136.324
Hokushinetsu	Nagano	36.039	138.112
Hokushinetsu	Nagano	35.711	137.907
Hokushinetsu	Nagano	35.544	137.843
Hokushinetsu	Toyama	36.973	137.607
Hokushinetsu	Toyama	36.683	137.109
Hokushinetsu	Toyama	36.671	137.031
Hokushinetsu	Toyama	36.664	137.268
Hokushinetsu	Toyama	36.628	136.874
Hokushinetsu	Fukui	36.170	136.294
Hokushinetsu	Fukui	35.973	136.203
Hokushinetsu	Fukui	35.825	136.203
Hokushinetsu	Fukui	35.603	136.150
Kii Peninsula	Mie	35.019	136.594
Kii Peninsula	Mie	34.868	136.417
Kii Peninsula	Mie	34.779	136.471
Kii Peninsula	Mie	34.613	136.462
Kii Peninsula	Mie	34.507	136.783
Kii Peninsula	Mie	34.493	136.696
Kii Peninsula	Mie	34.481	136.846
Kii Peninsula	Mie	34.465	136.572
Kii Peninsula	Mie	34.359	136.798
Kii Peninsula	Mie	34.344	136.641
Kii Peninsula	Mie	34.309	136.822
Kii Peninsula	Mie	34.279	136.510
Kii Peninsula	Mie	34.211	136.334
Kii Peninsula	Mie	34.101	136.199
Kii Peninsula	Mie	33.931	136.144
Kii Peninsula	Mie	33.804	136.043
Kii Peninsula	Wakayama	34.276	135.249

Kii Peninsula	Wakayama	34.037	135.201
Kii Peninsula	Wakayama	33.810	135.237
Kii Peninsula	Wakayama	33.681	135.358
Kii Peninsula	Wakayama	33.659	135.969
Kii Peninsula	Wakayama	33.592	135.934
Kii Peninsula	Wakayama	33.571	135.431
Kii Peninsula	Wakayama	33.506	135.599
Kii Peninsula	Wakayama	33.491	135.672
Kii Peninsula	Wakayama	33.488	135.794
Kii Peninsula	Wakayama	33.469	135.857
Kii Peninsula	Wakayama	33.454	135.760
Kansai	Shiga	35.356	136.307
Kansai	Shiga	35.217	136.284
Kansai	Shiga	35.079	136.192
Kansai	Shiga	34.998	135.869
Kansai	Shiga	34.976	135.943
Kansai	Kyoto	34.944	135.720
Kansai	Osaka	34.405	135.435
Chugoku	Tottori	35.500	133.784
Chugoku	Tottori	35.500	133.784
Chugoku	Tottori	35.500	133.784
Chugoku	Tottori	35.500	133.784
Chugoku	Tottori	35.500	133.784
Shikoku	Ehime	33.839	132.846
Kyushu	Kumamoto	32.939	131.114
Kyushu	Kumamoto	32.939	131.114
Kyushu	Kumamoto	32.939	131.114
Kyushu	Kumamoto	32.939	131.114
Kyushu	Saga	33.295	130.253
Kyushu	Saga	33.295	130.253
Kyushu	Saga	33.295	130.253
Kyushu	Saga	33.295	130.253

Kyushu	Saga	33.295	130.253
Kyushu	Saga	33.295	130.253
Kyushu	Saga	33.295	130.253
Kyushu	Kagoshima	31.752	130.197
Kyushu	Kagoshima	31.752	130.197
Kyushu	Kagoshima	31.752	130.197
Kyushu	Kagoshima	31.752	130.197
Kyushu	Kagoshima	31.752	130.197
Kyushu	Kagoshima	31.721	130.264
Amamiohshima	Kagoshima	28.501	129.690
Amamiohshima	Kagoshima	28.450	129.684
Amamiohshima	Kagoshima	28.440	129.710
Amamiohshima	Kagoshima	28.431	129.659
Amamiohshima	Kagoshima	28.423	129.643
Amamiohshima	Kagoshima	28.413	129.634
Amamiohshima	Kagoshima	28.403	129.586
Amamiohshima	Kagoshima	28.403	129.618
Amamiohshima	Kagoshima	28.392	129.504
Amamiohshima	Kagoshima	28.387	129.541
Amamiohshima	Kagoshima	28.385	129.495
Amamiohshima	Kagoshima	28.375	129.559
Amamiohshima	Kagoshima	28.334	129.502
Amamiohshima	Kagoshima	28.295	129.474
Amamiohshima	Kagoshima	28.290	129.444
Tokunoshima	Kagoshima	27.829	128.889
Tokunoshima	Kagoshima	27.824	128.959
Tokunoshima	Kagoshima	27.808	128.899
Tokunoshima	Kagoshima	27.804	128.993
Tokunoshima	Kagoshima	27.785	128.895
Tokunoshima	Kagoshima	27.775	128.912
Tokunoshima	Kagoshima	27.743	128.924
Tokunoshima	Kagoshima	27.713	128.907

Tokunoshima	Kagoshima	27.709	129.006
Tokunoshima	Kagoshima	27.686	128.923
Tokunoshima	Kagoshima	27.681	128.983
Tokunoshima	Kagoshima	27.673	128.953
Okinoerabujima	Kagoshima	27.434	128.704
Okinoerabujima	Kagoshima	27.425	128.679
Okinoerabujima	Kagoshima	27.409	128.635
Okinoerabujima	Kagoshima	27.398	128.662
Okinoerabujima	Kagoshima	27.396	128.621
Okinoerabujima	Kagoshima	27.393	128.574
Okinoerabujima	Kagoshima	27.386	128.542
Okinoerabujima	Kagoshima	27.362	128.536
Okinoerabujima	Kagoshima	27.356	128.616
Okinoerabujima	Kagoshima	27.332	128.574
Okinawa	Okinawa	26.665	127.927
Okinawa	Okinawa	26.665	127.927
Okinawa	Okinawa	26.665	127.927
Okinawa	Okinawa	26.665	127.927
Okinawa	Okinawa	26.665	127.927
Okinawa	Okinawa	26.241	127.742
Okinawa	Okinawa	26.237	127.728
Okinawa	Okinawa	26.223	127.697
Okinawa	Okinawa	26.220	127.727
Okinawa	Okinawa	26.217	127.691
Okinawa	Okinawa	26.212	127.676
Okinawa	Okinawa	26.202	127.654
Okinawa	Okinawa	26.198	127.659
Okinawa	Okinawa	26.195	127.659
Okinawa	Okinawa	26.193	127.660
Daitoujima	Okinawa	25.946	131.324
Daitoujima	Okinawa	25.946	131.324
Daitoujima	Okinawa	25.868	131.235

Daitoujima	Okinawa	25.864	131.224
Daitoujima	Okinawa	25.862	131.261
Daitoujima	Okinawa	25.857	131.233
Daitoujima	Okinawa	25.845	131.266
Daitoujima	Okinawa	25.843	131.221
Daitoujima	Okinawa	25.833	131.231
Daitoujima	Okinawa	25.829	131.220
Daitoujima	Okinawa	25.827	131.257
Daitoujima	Okinawa	25.823	131.221
Daitoujima	Okinawa	25.823	131.225
Daitoujima	Okinawa	25.818	131.236
Iriomotejima	Okinawa	24.424	123.794
Iriomotejima	Okinawa	24.396	123.803
Iriomotejima	Okinawa	24.396	123.803
Indoneshia	Java	8.009	110.286
Indoneshia	Java	7.998	110.249
Indoneshia	Java	7.895	110.543

Table S2. Pairwise genetic differentiation (F_{ST}) between populations in *Eleusine indica*

	Aogashima	Chubu	Chugoku	Hachijojima	Hokushinnetsu	Indoneshia	Iriomotejima	Kansai	Kanto	Kii Pen	Kouzushima	Kyushu	Miyakejima	Niijima	Nishinoshima	Ogasawara	Ohshima	Okinawa	Shikinejima	Tohoku	Tokai
Aogashima	0.000																				
Chubu	0.141	0.000																			
Chugoku	0.280	0.245	0.000																		
Hachijojima	0.146	0.100	0.205	0.000																	
Hokushinnetsu	0.162	0.084	0.197	0.128	0.000																
Indoneshia	0.317	0.270	0.443	0.211	0.258	0.000															
Iriomotejima	0.329	0.260	0.422	0.175	0.247	0.436	0.000														
Kansai	0.158	0.079	0.246	0.111	0.085	0.297	0.284	0.000													
Kanto	0.104	0.037	0.182	0.071	0.055	0.193	0.180	0.058	0.000												
Kii Pen	0.129	0.059	0.204	0.067	0.071	0.225	0.208	0.073	0.036	0.000											
Kouzushima	0.173	0.129	0.293	0.121	0.144	0.307	0.327	0.154	0.067	0.099	0.000										
Kyushu	0.150	0.071	0.269	0.091	0.120	0.249	0.258	0.110	0.047	0.068	0.135	0.000									
Miyakejima	0.142	0.097	0.238	0.099	0.110	0.262	0.277	0.118	0.061	0.090	0.087	0.122	0.000								
Niijima	0.106	0.169	0.359	0.156	0.175	0.368	0.365	0.188	0.111	0.141	0.187	0.175	0.154	0.000							
Nishinoshima	0.466	0.390	0.622	0.247	0.399	0.618	0.605	0.448	0.275	0.334	0.435	0.356	0.392	0.569	0.000						
Ogasawara	0.281	0.215	0.350	0.129	0.220	0.322	0.329	0.227	0.153	0.168	0.236	0.200	0.213	0.316	0.390	0.000					
Ohshima	0.172	0.105	0.310	0.131	0.125	0.343	0.325	0.130	0.063	0.077	0.135	0.113	0.141	0.217	0.505	0.265	0.000				
Okinawa	0.346	0.256	0.437	0.182	0.260	0.410	0.418	0.292	0.182	0.213	0.300	0.249	0.269	0.403	0.566	0.281	0.341	0.000			
Shikinejima	0.109	0.106	0.253	0.124	0.090	0.307	0.278	0.111	0.069	0.086	0.150	0.136	0.113	0.102	0.472	0.254	0.146	0.327	0.000		
Tohoku	0.125	0.046	0.191	0.089	0.045	0.221	0.212	0.061	0.018	0.045	0.083	0.065	0.073	0.133	0.322	0.177	0.078	0.214	0.077	0.000	
Tokai	0.111	0.034	0.174	0.064	0.054	0.197	0.178	0.049	0.020	0.026	0.089	0.043	0.076	0.119	0.280	0.145	0.066	0.183	0.072	0.024	0.000

Fig S1. The estimated log-likelihood (DLK2) and FST/FIS values for the most suitable value of *K* in PopCluster analysis.

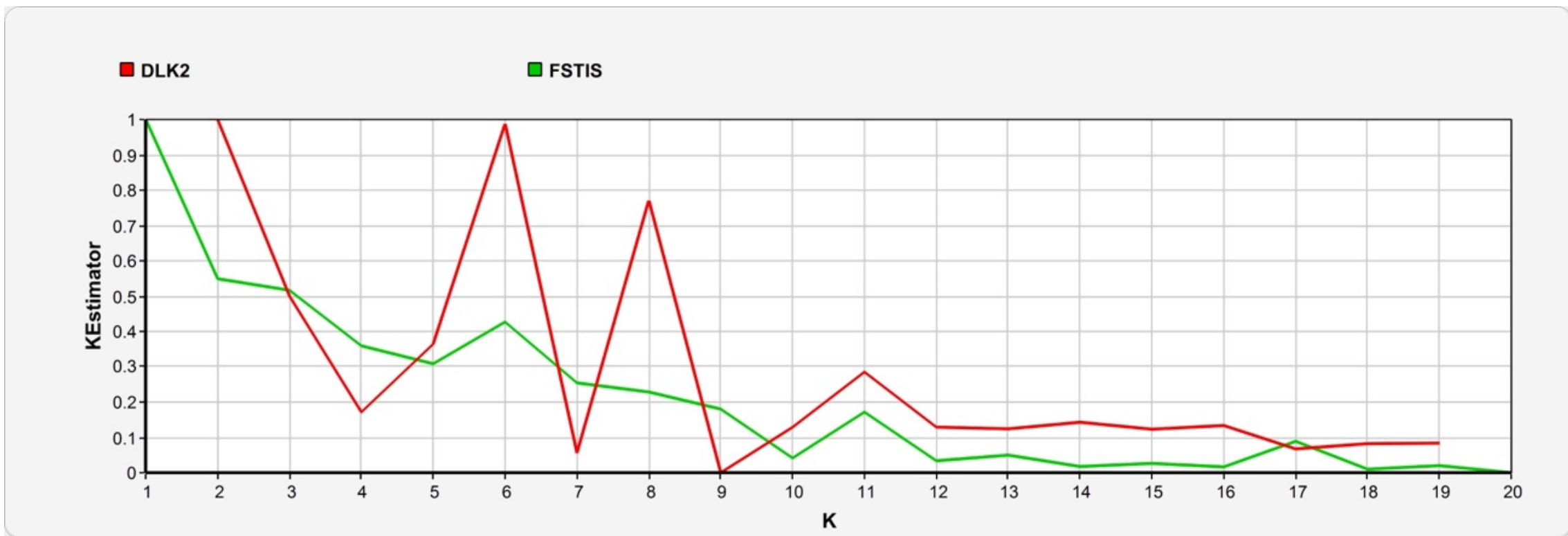

Fig S2. The results of PopCluster analysis of *K*=2-6.

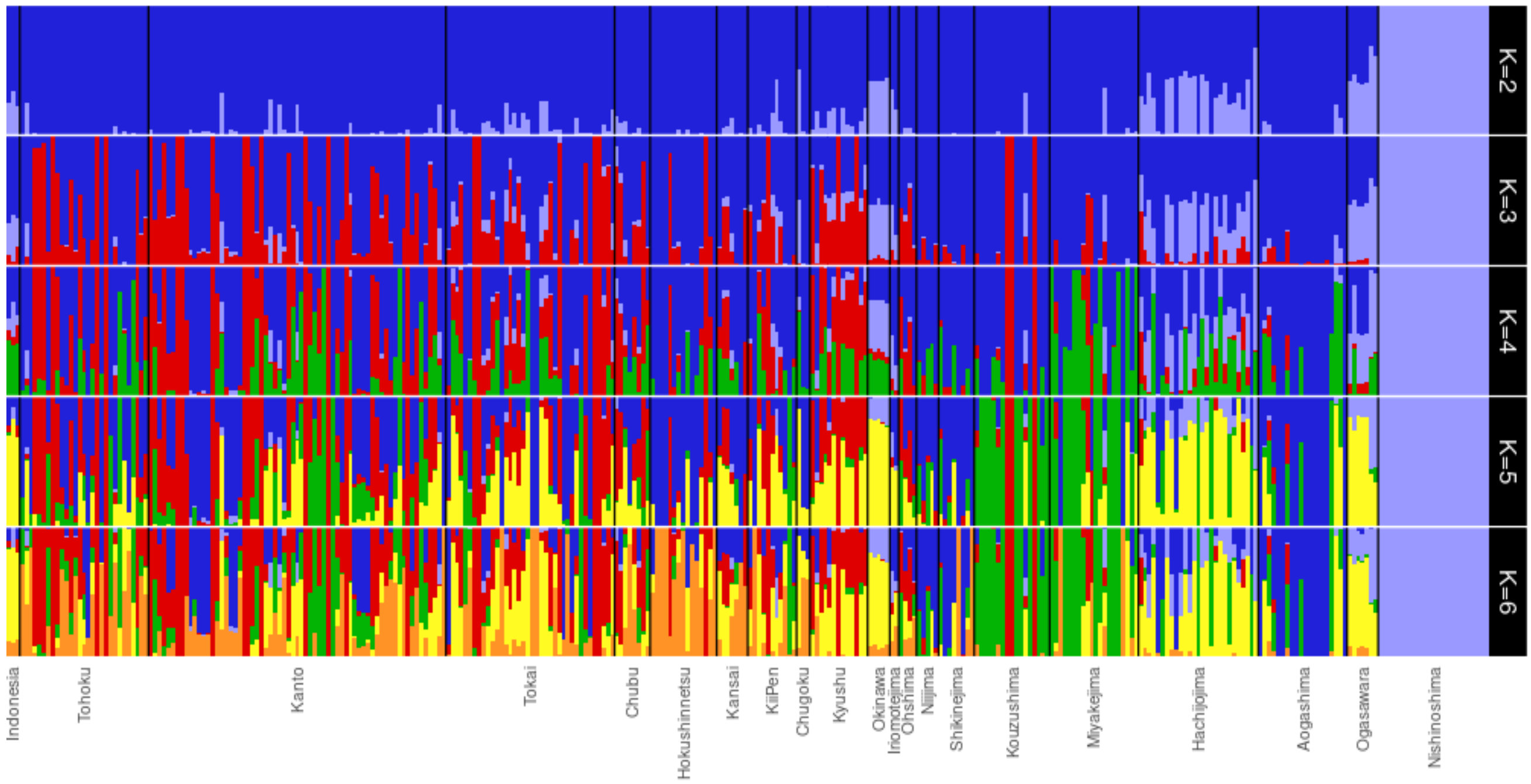

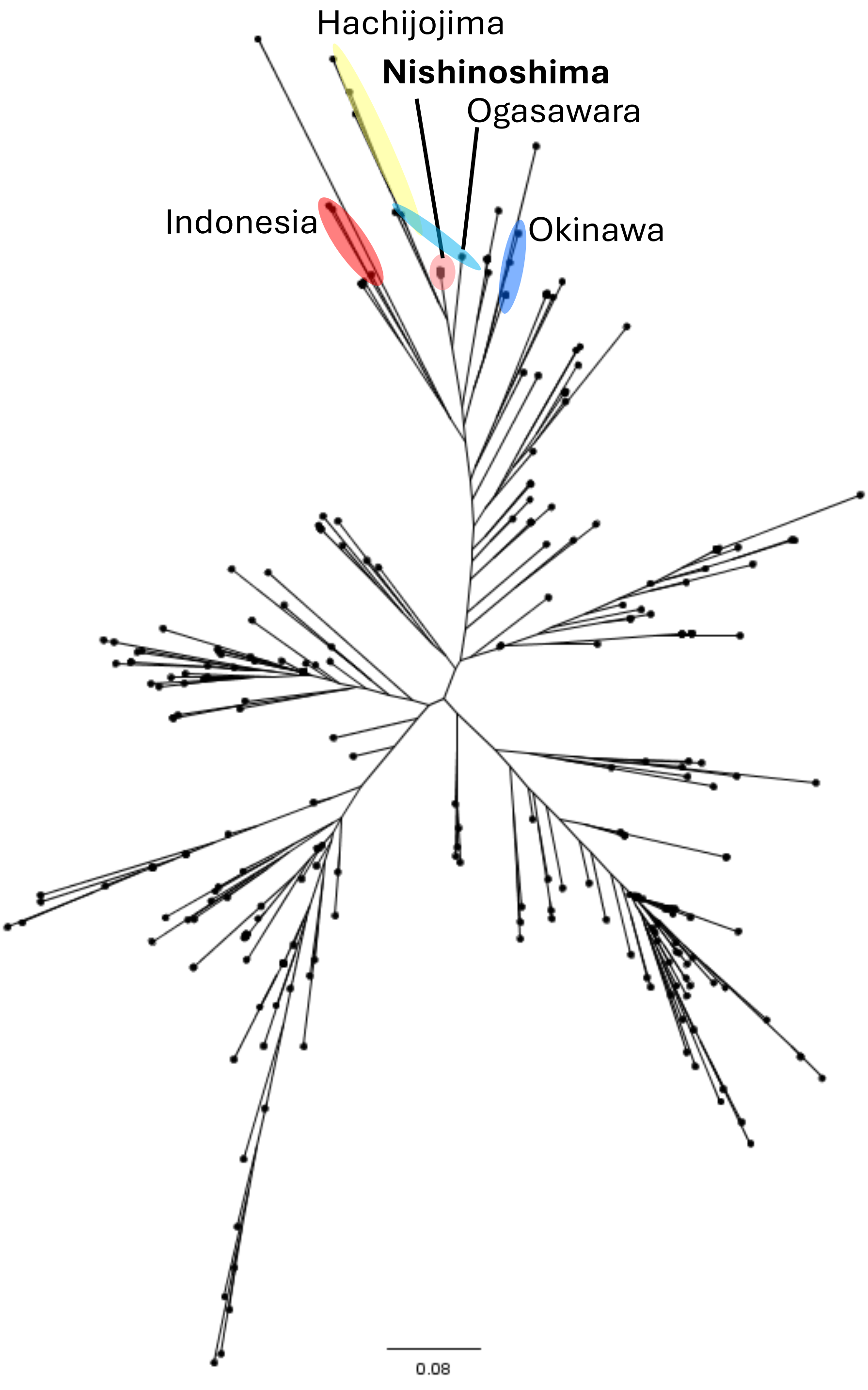

Fig. S3. The phylogenetic tree at individual level in *Eleusine indica*.

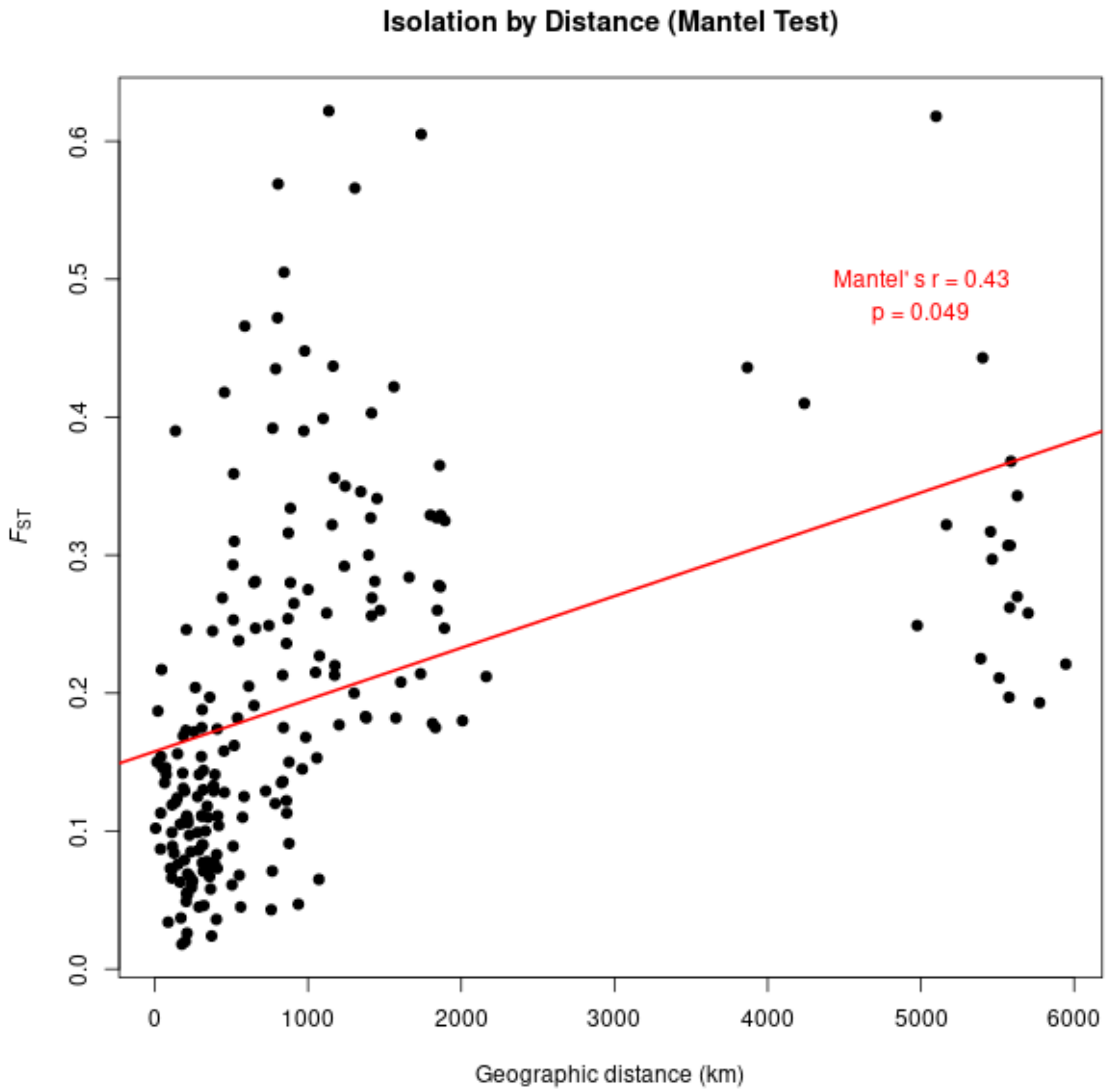

Fig. S4. A principal coordinate analysis (PCoA) plot based on pairwise F_{ST} at population level including Indonesia population.
